# Development and feasibility of stratified primary care physiotherapy integrated with eHealth in patients with neck and/or shoulder complaints: results of a mixed methods study

**DOI:** 10.1186/s12891-023-06272-6

**Published:** 2023-03-09

**Authors:** Mark L. van Tilburg, Corelien J.J. Kloek, Nadine E. Foster, Raymond W.J.G. Ostelo, Cindy Veenhof, J. Bart Staal, Martijn F. Pisters

**Affiliations:** 1 Expertise Center Healthy Urban Living, Research Group Innovation of Human Movement Care, HU University of Applied Sciences Utrecht, Heidelberglaan 7, 3584 CS Utrecht, The Netherlands; 2 Center for Physical Therapy Research and Innovation in Primary Care, Julius Health Care Centers, Utrecht, The Netherlands; 3 Primary Care Centre Versus Arthritis, School of Medicine, Keele University, Keele, UK; 4 Surgical Treatment And Rehabilitation Service (STARS), STARS Education and Research Alliance, The University of Queensland and Metro North Health, Brisbane, Australia; 5 Department of Health Sciences, Faculty of Science, VU University, Amsterdam Movement Sciences Research Institute, Amsterdam, The Netherlands; 6 Department of Epidemiology and Data Science, Amsterdam University Medical Center, Location VUmc, Amsterdam Movement Sciences Research Institute, Amsterdam, The Netherlands; 7 Department of Rehabilitation, Physiotherapy Science and Sports, UMC Utrecht Brain Center, University Medical Center Utrecht, Utrecht University, Utrecht, The Netherlands; 8 Musculoskeletal Rehabilitation Research Group, HAN University of Applied Sciences, Radboud University Medical Centre, Nijmegen, The Netherlands; 9 Radboud Institute for Health Sciences, IQ Healthcare, Radboud University Medical Center, Nijmegen, The Netherlands; 10 Research Group Empowering Healthy Behaviour, Department of Health Innovations and Technology, Fontys University of Applied Sciences, Eindhoven, The Netherlands

**Keywords:** Physiotherapy, Neck pain, Shoulder pain, Stratified care, Telehealth, eHealth, Blended care, Mixed methods, Feasibility

## Abstract

**Background:**

Providing individualized care based on the context and preferences of the patient is important. Knowledge on both prognostic risk stratification and blended eHealth care in musculoskeletal conditions is increasing and seems promising. Stratification can be used to match patients to the most optimal content and intensity of treatment as well as mode of treatment delivery (i.e. face-to-face or blended with eHealth). However, research on the integration of stratified and blended eHealth care with corresponding matched treatment options for patients with neck and/or shoulder complaints is lacking.

**Methods:**

This study was a mixed methods study comprising the development of matched treatment options, followed by an evaluation of the feasibility of the developed Stratified Blended Physiotherapy approach. In the first phase, three focus groups with physiotherapists and physiotherapy experts were conducted. The second phase investigated the feasibility (i.e. satisfaction, usability and experiences) of the Stratified Blended Physiotherapy approach for both physiotherapists and patients in a multicenter single-arm convergent parallel mixed methods feasibility study.

**Results:**

In the first phase, matched treatment options were developed for six patient subgroups. Recommendations for content and intensity of physiotherapy were matched to the patient’s risk of persistent disabling pain (using the Keele STarT MSK Tool: low/medium/high risk). In addition, selection of mode of treatment delivery was matched to the patient’s suitability for blended care (using the Dutch Blended Physiotherapy Checklist: yes/no). A paper-based workbook and e-Exercise app modules were developed as two different mode of treatment delivery options, to support physiotherapists. Feasibility was evaluated in the second phase. Physiotherapists and patients were mildly satisfied with the new approach. Usability of the physiotherapist dashboard to set up the e-Exercise app was considered ‘OK’ by physiotherapists. Patients considered the e-Exercise app to be of ‘best imaginable’ usability. The paper-based workbook was not used.

**Conclusion:**

Results of the focus groups led to the development of matched treatment options. Results of the feasibility study showed experiences with integrating stratified and blended eHealth care and have informed amendments to the Stratified Blended Physiotherapy approach for patients with neck and/or shoulder complaints ready to use within a future cluster randomized trial.

**Supplementary Information:**

The online version contains supplementary material available at 10.1186/s12891-023-06272-6.

## Background

Musculoskeletal (MSK) pain conditions are substantial contributors to global disability [1]. Two frequently occurring MSK pain presentations are neck and shoulder complaints [1, 2]. Recently, key recommendations for high-quality primary care for MSK conditions were published [3]. Primary care physiotherapists can support patients with neck and shoulder complaints in their recovery by providing treatment that addresses physical activity and exercise, by assessing psychosocial factors and by educating patients about their condition and management options. Additionally, primary care need to be patient-centered, which means providing individualized care based on the context and preferences of the patient [3]. To meet these recommendations, there is a need for guidance about how to offer patient-centered care.

To guide physiotherapists in achieving patient-centered care, stratified care is one possible approach [4]. Stratified care has two components; firstly the use of a tool to identify subgroups of patients and secondly matching treatments to patients in each subgroup [4]. A well-known and extensively studied example of stratified care for patients with low back pain (STarT Back) is based on identifying patients’ risk of persistent pain and then matching subgroups of patients (at low, medium and high risk) to the most appropriate treatment [5, 6]. Besides risk stratification, patient-centered care can also be based on patients’ suitability for different modes of treatment delivery, such as face-to-face physiotherapy or care that is blended with digital guidance. Despite the potential for these two approaches to individualize care for MSK patients with neck and/or shoulder complaints, no studies have yet combined them and tested their feasibility or effectiveness.

A first approach to stratified care in patients with neck and shoulder complaints is to match content and intensity of physiotherapy treatment to the patient’s risk of persistent disabling pain, using the Keele STarT MSK Tool [5, 6]. The Keele STarT MSK Tool can be used to classify patients as either low, medium or high risk of persistent disabling pain, which can guide physiotherapists in their clinical reasoning to match the patient to the most appropriate content and intensity of physiotherapy treatment [7, 8]. The Keele STarT MSK Tool consists of ten items assessing the patient’s pain and coping, impact of pain, function and disability and comorbidity and has shown good predictive and discriminative ability among UK primary care patients [9, 10]. The Dutch version of the Keele STarT MSK Tool has shown sufficient to good validity and reliability among Dutch primary care patients with MSK pain [11]. Recently, recommended matched treatment options were developed for primary care providers in the treatment of MSK pain and for physiotherapists in the treatment of patients with low back pain [12, 13]. However, no physiotherapy-specific matched treatment options have yet been agreed for patients with neck and/or shoulder complaints.

A second approach to individualize care is to match the mode of physiotherapy treatment delivery to the patient’s suitability for blended treatment. With the introduction of new technologies within physiotherapy, generally two modes of delivery of physiotherapy treatment can be distinguished: a traditional treatment consisting of face to face physiotherapy guidance, and a blended treatment in which physiotherapy sessions are integrated with technology such as a smartphone application to help patients manage their MSK pain independently, in the home environment [14–16]. New technologies have the potential to assist physiotherapists in supporting patients’ adherence to recommended physical activity behavior, exercises and management options and provide visual insight in patient progress. Since not every patient has the required skills and motivation for blended care, physiotherapists may need to first identify whether blended care is appropriate for the patient [17, 18]. This can be done by using the Dutch Blended Physiotherapy Checklist [19]. This is a clinical decision aid for the physiotherapist in deciding upon suitability for blended physiotherapy, and contains items to assess the patients’ motivation, safety, equipment, digital skills, health literacy, self-management, time, and financial situation [19]. In order to prevent health care inequalities, patients that are not suitable for blended care are likely best offered offline alternatives.

Despite tools being available to identify patients’ (i) risk of persistent disabling pain (Keele STarT MSK Tool) and (ii) suitability for blended physiotherapy care, no study has yet combined these approaches to individualize primary care nor developed suitable matched treatments for each patient subgroup. Therefore, this study aimed to: (1) develop physiotherapy-specific matched treatment options as part of a new Stratified Blended Physiotherapy approach for patients with neck and/or shoulder complaints that matches the values of end-users; and (2) investigate feasibility of the Stratified Blended Physiotherapy approach for patients with neck and/or shoulder complaints prior to determining its effectiveness in a future cluster randomized trial [20].

## Methods

### Study design

This study was a two phase, mixed methods study comprising first the development of matched treatment options, followed by evaluating feasibility of the developed Stratified Blended Physiotherapy approach.

#### Phase 1 - development of matched treatment options

The first phase comprised the development of physiotherapy-specific matched treatment options. To support the agreement about matched treatment options, three focus groups were conducted. An exploratory, descriptive phenomenological approach was used, with thematic analyses.

#### Phase 2 - feasibility of the stratified blended physiotherapy approach

The second phase investigated the feasibility of the developed Stratified Blended Physiotherapy approach for patients with neck and/or shoulder complaints. Feasibility was evaluated in a multicenter single-arm convergent parallel mixed methods study. Both quantitative and qualitative data were collected independently and analyzed in parallel. These quantitative and qualitative results were compared to look for patterns and contradictions to identify possible improvements to the Stratified Blended Physiotherapy approach.

### Participants

#### Phase 1 - development of matched treatment options

Twenty-five stakeholders with expertise in the field of eHealth, stratified care and neck and/or shoulder complaints were invited to one of three focus groups, including physiotherapists, researchers, eHealth entrepreneurs, teachers and physiotherapy policy officers. All stakeholders had a background in physiotherapy. Participants were identified from the authors’ professional networks, and invited by email.

#### Phase 2 - feasibility of the stratified blended physiotherapy approach

Physiotherapists

Twenty-eight physiotherapists working in primary care were invited to participate in the feasibility study. These physiotherapists were also from the authors’ professional network, and were different to those in the first phase. Physiotherapists were eligible to participate if they treated at least four new patients with neck and/or shoulder complaints per month.

Patients

Patients with sufficient mastery of the Dutch language were eligible for participation if they had experience of subacromial pain syndrome, biceps tendinosis, shoulder instability or non-specific MSK pain of the neck and/or shoulder (not caused by acute trauma (fracture or rupture) or by systemic disease) [21, 22]. Patients were excluded if their neck and/or shoulder disorder was caused by a specific pathology (e.g. shoulder pain with loss of active and passive range of motion [frozen shoulder], vertebral fracture, tendon rupture, Parkinson’s disease, herniated nucleus pulposus, cervical stenosis), except for subacromial pain syndrome, biceps tendinosis and shoulder instability.

### Data collection

#### Phase 1 - development of matched treatment options

Three focus groups were conducted in September and October 2018. The focus groups were facilitated by authors CK and MvT and lasted approximately one hour. In all three focus groups, a facilitator provided a study overview and a brief overview of a similar recently developed blended intervention for a different patient group (e-Exercise Low Back Pain [12, 16, 23]) that could be used as the basis of the Stratified Blended Physiotherapy approach. Topics discussed in the focus groups were: design of the Stratified Blended Physiotherapy approach, experiences or expectations of stratified care, experiences or expectations of blended care, including requirements of the app and offline alternative, and content and intensity of matched treatments.

#### Phase 2 - feasibility of the stratified blended physiotherapy approach

Participating physiotherapists received a four-hour training session on how to integrate the Stratified Blended Physiotherapy approach into their daily practice and the study procedures. Physiotherapists were asked to aim for the inclusion of at least two patients. Recruitment of patients for the feasibility study lasted from April 2019 until June 2019. During the initial registration, eligible patients were verbally informed about the study and invited to participate in the data collection by the participating physiotherapy practice secretariat. Every potentially eligible patient who was not invited by the physiotherapy practice secretariat, was invited by participating physiotherapists. If the patient agreed to participate in the data collection, contact details were sent to the researcher via a secure messenger service. Thereafter, the researcher sent an information letter to the patient by email. Subsequently, the researcher contacted the patient by phone to check whether the patient read an understood the information letter. If so, the researcher screened the eligibility criteria. After informing the participant about the study and the assessment of their eligibility, an informed consent form was sent to the participant by mail. After consenting to participate, the patient was asked to send the signed informed consent form back to the research team. An extra screening of the eligibility criteria was performed by the physiotherapist during the first physiotherapy session.

Quantitative data

A digital questionnaire was sent to the patients three months after the first physiotherapy session. There was no maximum number of patients that could be included per physiotherapist. Physiotherapists received a questionnaire at the end of the patient recruitment period. Quantitative data collection in the feasibility study consisted of three measures:

- Patients’ satisfaction with treatment was measured with the Global Perceived Effect – Dutch Version scale (GPE-DV) [24]. The GPE-DV is a 2-item questionnaire about patient satisfaction with the treatment (1: ‘Extremely satisfied’ to 7: ‘Extremely dissatisfied’) and experienced effect (1: ‘Much improved’ to 7: ‘Worse than ever’) on a 7-point Likert scale. Physiotherapists satisfaction with treatment according to the Stratified Blended Physiotherapy approach was also measured with the same 7-point Likert scale.
- Whether a patient received a smartphone app or paper-based workbook and subsequently patient willingness to recommend either the smartphone app or paper-based workbook was evaluated using the Net Promoter Score (NPS) [25]. The NPS is a 1-item question about patient satisfaction (0: ‘not at all likely’ to 10: ‘extremely likely’) on a 11-point Likert scale. As suggested in previous research on usage of the NPS in health care settings, only the mean or median NPS was reported, rather than also NPS categorization [26].
- Patients’ experienced usability of the smartphone app and physiotherapists’ experienced usability of the physiotherapist dashboard was evaluated with the System Usability Scale (SUS) [27]. The total SUS score ranged from 0 to 100 (0-12.5: ‘worst imaginable’ to 85.6–100: ‘best imaginable’) [28].

Qualitative data

Qualitative data collection consisted of semi-structured interviews with both patients and physiotherapists. All physiotherapists and patients were consecutively invited by e-mail to participate in a semi-structured interview. Semi-structured interviews were held after patients completed the intervention. An exploratory, descriptive phenomenological approach was used, with thematic analyses [29, 30]. The interviews were individually conducted by MvT in participants’ homes or by phone and lasted approximately 30 min. Besides the researcher and participant, no one else was present during the interview. The researcher who conducted the interviews was trained in performing interviews and had no treatment relationship with the patients. Topic guides were developed by MvT and CK and discussed by the authors to get feedback, and focused on three main areas based on the determinants of innovation described by Fleuren et al.: characteristics of the participant, intervention and organization [31]. Interview topic guides were pilot tested between MvT and CK and are provided in Appendix 1.

### Analysis

Descriptive statistics were used to describe participant characteristics. All quantitative data analyses were performed using SPSS Version 25 (IBM Corp., Armonk, NY, USA). All qualitative data were audio-recorded and transcribed verbatim by MvT. Using data from the first focus group, first three of nine patient and first three of eight physiotherapist interviews, MvT and CK independently identified codes, grouped them into meaningful categories and subsequently categorized them into main themes using ATLAS.ti 9 for Windows. Codes and main themes from these interviews were discussed between MvT and CK until consensus was reached. After the consensus meeting, MvT independently analyzed the rest of the interviews and focus groups. Transcripts and findings were not returned to participants for comments or corrections and field notes were not made, because data were felt to be straightforward and unlikely to be misinterpreted.

## Results

### Phase 1 - development of matched treatment options

Three focus groups were conducted with physiotherapists and physiotherapy experts. Focus group participant characteristics can be found in Table 1.

**Table 1:** Focus group participant characteristics

	*Focus group 1*	*Focus group 2*	*Focus group 3*
Number of participants	5	5	7
Sex, female, *n* (%)	2 (40)	1 (20)	2 (29)
Age in years, *median* (IQR)	34 (22)	36 (19)	36 (16)
Work background, *n* (%)* *Physiotherapy* *Researcher* *eHealth entrepreneur* *Educator* *Policy officer*	5 (100) 1 (20) 0 (0) 1 (20) 0 (0)	5 (100) 1 (20) (0) 1 (20) (0)	7 (100) 4 (57) 1 (14) 4 (57) 2 (29)
Masters education or higher, yes, *n* (%)	4 (80)	3 (60)	7 (100)

*Columns add to percentages that exceed 100%, because participants might have multiple work backgrounds

Main themes and related codes are described below. A visual representation of the developed Stratified Blended Physiotherapy approach, including the developed matched treatments, is presented in Table 2.

#### Content and intensity of physiotherapy

*Content and intensity of physiotherapy sessions.* According to the stakeholders, a main structure similar to the previously developed ‘e-Exercise Low Back Pain intervention’ was mentioned to be suitable: i.e. an information, exercise and physical activity module [12]. As for the intensity of treatment, stakeholders mentioned that the number of sessions per risk subgroup need to be somewhat similar to the e-Exercise Low Back Pain intervention (Low risk: 2 sessions, medium risk: max. 8 sessions, high risk: max. 12 sessions), but instead of just stating a maximum number, they recommended a range of treatment sessions per risk subgroup (minimum to maximum number of sessions). Additionally, participants suggested to add a timeframe for physiotherapy session delivery of 3 weeks for patients at low risk and 12 weeks for those at medium and high risk, corresponding with the Dutch physiotherapy clinical guideline for neck pain [32].

*Adaptability and physiotherapists’ behavior change.* Stakeholder focus groups highlights perceived added value of using patients’ risk of persistent disabling pain (prognosis), instead of diagnosis alone, to inform treatment content and intensity. The aim of stratified care is to encourage physiotherapists to offer more treatment sessions to patients at high risk whilst offering fewer treatments to those at low risk.

*Look, a great deal of patients – at least with low risk – are overtreated. And in patients at high risk, the focus of the treatment is chosen incorrectly. I think we will do reasonably well with six treatment sessions. And I do think that therapists should be more aware of their interventions and they should reflect critically on the applied number of treatment sessions. [Physiotherapist 3, focus group 1]*

However, participants stated that the Stratified Blended Physiotherapy approach should not be a strict protocol, but rather be flexible so that physiotherapists can personalize the components based on their clinical reasoning and patients’ expectations.

*Somehow, you want to prevent it from turning into some kind of assembly line work, and that the therapist no longer thinks about the kind of care that they provide*. *[Physiotherapist 6, focus group 2]*

Additionally, there were some concerns that not every physiotherapist might be competent to treat patients at high risk, but nevertheless participants stated it needs to be up to physiotherapists to decide if they feel competent enough to manage these patients.

#### Mode of Treatment delivery

*Added value.* The determination of patients’ suitability for blended physiotherapy seemed relevant, according to stakeholders. They mentioned that potential benefits of integrating an app or paper-based workbook within treatment sessions might be the contribution to supporting patient self-management skills and improving adherence to exercise and physical activity recommendations. Interviewees’ proposed this might contribute to changing some patients’ expectations about physiotherapy away from a passive to a more active approach.

*Readiness for implementation.* Focus group data highlighted the perceived importance of physiotherapists being familiar with blended care as a treatment option and learning about the potential benefits of it for themselves and patients. Additionally, the physiotherapist’s digital skills, experience with blended care and the way in which the app is introduced and integrated within the treatment were mentioned to be important factors for successful implementation.

*Therapeutic alliance.* Participants emphasized the importance of the physiotherapist-patient relationship for successful integration of e-Exercise and treatment sessions.

*We all know that non-verbal communication is very important. That you and the patient can evaluate whether or not the treatment progress goes as expected. [Physiotherapist 10, focus group 2]*

#### Practical tool – e-Exercise app functionalities

*Content of the information module.* Work-related factors were mentioned to be important to explicitly address, where relevant for individual patients. Participants reported that the emphasis of the information could better be on patient activities and abilities rather than pain, that information needs to be easy to understand and practically applicable, and that every information theme needs to contain a question for the patient, so that the physiotherapist can check whether the patient has read and understood the information.

*What you often see in information provision in digital applications is that information is too complicated or too difficult to practically apply. [Physiotherapist 13, focus group 3]*

*Content of the exercise module.* Participants stated that, preferably, there needs to be a preselection of video-supported exercises in the app, so that the physiotherapist can delete unnecessary exercises and add new specific exercises. Participants also perceived the value of being able to add personal notes to the exercise program, and being able to video-record an individual patient and upload it to that patients’ individual exercise program in the app.

*Content of the physical activity module.* Since physiotherapists and patients stated the physical activity module and the option to include graded activity principles were not applicable to every patient with neck and/or shoulder complaints, participants suggested that the graded activity principles (behaviorally oriented treatment) can be optional. Additionally, a reminder function in the app to change postures was suggested as an alternative.

*I think it very much depends on the patient. If people are inactive, then I think it’s a very useful thing to do. But of course you also see people who are already active enough, or perhaps do too much. Anyway, if you can check the physical activity module on or off, then you can personalize that per patient. [Physiotherapist 7, focus group 2]*

*User-friendliness.* Recommendations about the interface of the app were that it needs not be too complex (since complex features would be unlikely to be used), that a communication function was unnecessary, and that the ability to add personal notes within the various modules might be useful. For the implementation, physiotherapists mentioned that it would be useful if the electronic health record (EHR) system could guide the physiotherapist through every step of the Stratified Blended Physiotherapy approach, including the app modules. Furthermore, therapists reported the app needs to be integrated, or communicate, with the most frequently used EHR systems.

*You can develop something with all kinds of nice features, but they will not be used if it’s - only even a little - too complex. [Physiotherapist 13, focus group 3]*

#### Practical tool – paper-based workbook

*Relative changes and advantages compared to e-Exercise.* Physiotherapists stated that content of the paper-based workbook needs to be similar to the content of the app. In contrast to the app since there can be no content matching of information based on risk subgroup, type of complaints, or work status, there should be only one workbook for all patients. Physiotherapists mentioned that an advantage of the paper-based workbook is that it can easily be personalized by making notes in texts, assignments or exercises.

*The fact that you tick chapters makes it personal. Then they will also read the book immediately, which they do not do if you just give it. So, just scratching something into it makes it personal. [Physiotherapist 3, focus group 1]*

### Developed version of the stratified blended physiotherapy approach, including matched treatments

A visual representation of the developed Stratified Blended Physiotherapy approach, including the matched treatments, is presented in Table 2. Physiotherapists were provided with the two tools, one to identify patients in different risk subgroups and one to identify those who were suitable (or unsuitable) for blended care delivery. Theoretically, that led to six matched treatment subgroups (suitable for blended care in combination with low, medium or high risk and unsuitable for blended care in combination with low, medium or high risk). If considered suitable for blended care, the patient was then to receive a blended physiotherapy treatment (e-Exercise), in which a smartphone app with personalized information, exercises and physical activity modules was an integral part of physiotherapy treatment. The e-Exercise app module is integrated within one of the largest electronic health record systems (EHRs) of the Netherlands. Physiotherapists had access to a web based dashboard to set up, adjust and monitor the progress of the three modules. Content of the e-Exercise modules differed per risk subgroup. Several behavior change techniques were incorporated within the e-Exercise modules: framing/reframing and focus on past success, restructuring the social environment, reduce negative emotions, social incentive, social reward, credible source, graded tasks, generalization of target behavior, habit formation, behavior substitution, (reduce) prompts/cues, social comparison, demonstration of the behavior, information about health and emotion consequences, instruction on how to perform the behavior, social support, self-monitoring of behavior, feedback on behavior, goal setting (behavior and outcome) and, review behavior goals [33]. As an example, the e-Exercise module asked patients to plan physical activities and exercises, and patients’ could monitor their treatment progress (track concluded assignments in the information module, track performed exercise in the exercise module and track number minutes of their physical activity in the physical activity module). After concluding physiotherapy treatment, agreements made between physiotherapist and patient were noted and, in the app, six messages were sent over 12 weeks to remind patient of the lessons learnt. Additionally, the e-Exercise information module remained available for patients to reread or review at any time, without an end-date. If patients were considered to be unsuitable for blended care, a paper-based workbook with similar content was to be integrated within physiotherapy treatment. However, the paper-based workbook did not contain the video’s, animations, reminders, tailored feedback, and content matching on type of complaints, risk subgroup, and work status. The researchers collaborated with eHealth entrepreneur ‘The Health Train BV’, because they have a commercially available smartphone app with video-supported exercises in which the e-Exercise module for patients with neck and/or shoulder complaints could be integrated (www.mijnzorgapp.com). A photo of a person using one of the e-Exercise modules is provided in Appendix 1.

**Table 2:** Overview of the Stratified Blended Physiotherapy approach, developed in phase 1 of this study

**Step 1. Stratification**			
**Content and intensity of treatment** Is to be matched to the patient’s risk of persistent disabling pain (low, medium or high, assessed with the Keele STarT MSK Tool)			
**Mode of treatment delivery** Is to be matched to the patient’s suitability for blended care (yes or no, assessed with the Dutch Blended Physiotherapy Checklist by the physiotherapist)			
**Step 2. Matched treatment options (described per risk profile)**			
	**Low risk**	**Medium risk**	**High risk**
*Physiotherapy sessions (either face-to-face or video consults)*			
**Aim**	Improvement of pain and disability		
**Intensity of physiotherapy**	3–4 sessions (over 3 weeks)	6–9 sessions (over 12 weeks)	8–12 sessions (over 12 weeks)
**Content**	Reassurance, provide information on neck/shoulder complaints, possible causes, self-management options and the importance of adequate physical activity/exercise behavior	Similar to low risk with the optional addition: provide passive or active joint mobilization techniques, in combination with functional exercise therapy	Similar to medium risk with the optional addition: address patient’s specific physical and psychosocial obstacles to recovery, using a combination of physical and psychological approaches, including pain education
**Integration of e-Exercise or paper-based workbook**	Motivate to read information modules and do home-based exercises independently	Per session evaluation of progress with e-Exercise app or paper-based workbook to optimize physiotherapy treatment	
**Evaluation**	In the final session: evaluate the progress and give recommendations to prevent recurrent episodes of neck/shoulder complaints and maintain or improve the physical activity level		
*Patient’s home environment; e-Exercise app or paper-based workbook*			
**Information module**	3 weekly varying information themes, including assignments to stimulate patient self-reflection	12 weekly varying information themes, including assignments to stimulate patient self-reflection, about the etiology of neck/shoulder complaints, physical activity, patient experiences, pain management, and psychosocial factors related to neck/shoulder complaints. The information and order of the information provided differs per risk subgroup and working status	
**Exercise module**	3–4 personalized exercises to fit the patient’s specific functional status		
**Physical activity module**	Not applicable	The patient chooses one physical activity and sets a goal to maintain or enhance the level of that physical activity. Optionally, a ‘graded activity’ functionality can be activated.	The patient chooses one physical activity and sets a goal to enhance the level of that physical activity, by using a ‘graded activity’ functionality.

### Phase 2 - findings of the feasibility study

Eight physiotherapists invited 23 eligible patients. Of these patients, 13 signed informed consent forms and were enrolled in the study and the rest declined to participate after reading the information letter. Eight physiotherapists and 13 patients completed the follow-up questionnaire. No patients were lost to follow-up and two physiotherapists were lost to follow-up due to self-reported lack of experience with the Stratified Blended Physiotherapy approach and therefore were not willing to participate in the follow-up data collection. Physiotherapist and patient characteristics are presented in Tables 3 and 4, respectively.

**Table 3:** Feasibility study physiotherapist characteristics

Number of physiotherapists	8
Sex, female, *n* (%)	5 (63)
Age in years, *median* (IQR)	42 (19)
Specialization, *n* (%) *Manual therapy* *No relevant Master’s degree*	5 (63) 3 (37)
Experience with blended or stratified physiotherapy, *n* (%) *Yes, stratified physiotherapy* *Yes, blended physiotherapy* *Yes, both* *No, neither*	4 (50) 2 (25) 0 (0) 2 (25)

**Table 4:** Feasibility study patient characteristics

	*Quantitative analysis*	*Qualitative analysis*
Number of patients	13	9
Sex, female, *n* (%)	6 (46)	3 (33)
Age in years, *median* (IQR)	55 (11)	57 (12)
Risk subgroup (STarT MSK Tool), *n* (%) *Low risk* *Medium risk* *High risk*	8 (61) 4 (31) 1 (8)	5 (56) 3 (33) 1 (11)

### Phase 2 - quantitative results

Physiotherapists’ satisfaction with delivery of the new approach was scored as ‘a little satisfied’ (median = 3; IQR = 1; n = 8). Physiotherapists’ experienced usability of the physiotherapist web based dashboard of the app was 40 (median; IQR = 9.4; n = 8), which is considered as ‘OK’ usability [28].

Patients’ rating of global improvement at the end of treatment was scored as ‘a little improved’ (median = 3; IQR = 2; n = 13). Patients’ satisfaction with treatment was scored as ‘a little satisfied’ (median = 3; IQR = 2; n = 13). Patients’ willingness to recommend the app was 9 (median; IQR = 4; n = 11). No patients received the paper-based workbook, because there were no patients considered unsuitable for e-Exercise by physiotherapists. Patients’ experienced usability of the developed app was 87.5 (median; IQR = 30; n = 11), which is considered as ‘best imaginable’ usability.

### Phase 2 - qualitative results

All 8 physiotherapists and 9 of the 13 invited patients who completed the follow-up questionnaires were interviewed. Four patients did not want to participate in an interview. Results were structured according to the main themes.

#### Content and intensity of physiotherapy

*Added values.* According to physiotherapists, the Keele STarT MSK Tool was experienced as helpful to decision-making about patient subgroups.

*Current physiotherapist guidelines also provide us with treatment profiles, but now I have more of a fixed tool that gives handles for selecting a treatment profile. [Physiotherapist 20]*

*Content and intensity of physiotherapy sessions.* Physiotherapists suggested matched treatment content and intensity recommendations for each risk subgroup were too extensive for patients at low risk with a first episode of neck/shoulder pain, but adequate for patients with a recurrence. The content and intensity of physiotherapy treatment was reported to potentially have add value as preventive measure for patients at low risk, as one physiotherapist explained:

There are many low risk patients that can learn from it, also preventively. [Physiotherapist 22

Physiotherapists pointed out that the content and intensity of treatment for patients at medium and high risk was of added value, because the treatment was more extensive, mainly in the way of providing (pain) education.

*Adaptability.* Physiotherapists liked to be able to deviate from the recommended matched treatments, based on their clinical reasoning and patients’ expectations. Additionally, one physiotherapist explained that, if the patient was open to it, she referred high risk patients to a colleague that was more experienced in psychosomatic care. Physiotherapists agreed that referral of patients at high risk to a physiotherapist with more knowledge in this area can better not be standard practice, but up to the physiotherapist in consultation with the patient.

#### Mode of treatment delivery

*Added values.* Physiotherapists said that they did not really use the second tool to guide individualization of care, the Dutch Checklist Blended Physiotherapy. Physiotherapists explained they were able to decide suitability for blended physiotherapy just using their clinical expertise. The tool however was thought to potentially be beneficial as a learning instrument in the case where the physiotherapy is unsure, as one physiotherapist explained:

*I believe it can be used when in doubt, but you don’t have to use it standardly. [Physiotherapist 25]*

*Physiotherapists’ training.* Instead of the four-hour training, physiotherapists mentioned a physiotherapists’ training of two half-days in the Stratified Blended Physiotherapy approach, with at least a week between sessions, would be essential to gain experience with e-Exercise and successfully integrate the app with physiotherapy treatment. However, the need for training before being able to deliver the Stratified Blended Physiotherapy approach was experienced as a barrier to referring patients to colleagues that have not participated in the training.

*Readiness for implementation.* If a patient was suitable for blended physiotherapy, physiotherapists reported they had to enthuse patients before integrating an app within physiotherapy treatment, however when the physiotherapist was not yet fully motivated or competent in using the app, that was experienced as a challenge.

*Compatibility with work routine.* Although they did not use it, physiotherapists stated that the paper-based workbook was a useful alternative to the app. As for the app, time investment in setting up, explaining and integrating the app for patients at low risk of persistent disabling pain was mentioned to be too long. Physiotherapists were not convinced that patients wanted a relatively large part of the treatment session to be spent on the app. However, as one patient explained:

*The physiotherapist thought it was too much. However, I thought, well, you know, if it is necessary, it is necessary’. [Patient 6]* Another patients also noted: *‘Just by involving me, like: we are going to add this exercise to the app. That was pleasant.’ [Patient 2]*

*Adaptability.* Patients and physiotherapists pointed out that they liked to be flexible in terms of which app module might be the most important for a specific patient and thus this required more attention within treatment sessions.

#### App functionalities

*Added values.* Patients were positive about using the app, as one patient noted:

*I am convinced that, thanks to the app, I did my exercises more often and I recovered more quickly. [Patient 6]*

Even a patient who stated they were not very digitally skilled, noted that the app was *‘nice and worked as a carrot on a stick’ [Patient 8].* Additional added values reported by patients were that they could work on their recovery at home, in their own time and place. One patient was planning to use the app again after conclusion of the physiotherapy treatment, the moment their symptoms might recur.

*Adaptability.* Currently, e-Exercise is integrated within a commercially available smartphone app with video-supported exercises. Physiotherapists that used, or had experience with, a different provider of video-supported exercises experienced the use of a new, and in some cases an additional, provider as a barrier to adoption.

*Especially, I would like to choose between various app providers to integrate e-Exercise. [Physiotherapist 21]*

Additionally, the app is integrated within one of the largest EHR systems of the Netherlands. However, physiotherapists with other EHR systems used a stand-alone version of e-Exercise and therefore had to use two independent systems. This was experienced as a barrier to the adoption of e-Exercise.

*So we run our own system, the dashboard, and then also the exercises portal. That makes me think ‘waah’. [Physiotherapist 20]*

*Design quality of the physiotherapist dashboard.* Physiotherapists mentioned the web based dashboard required to set up, adjust and monitor the progress of the three modules was lacking a ‘cockpit functionality’ with an overview of active patients and their progress. They also stated the dashboard lacked a logical workflow. Physiotherapists experienced these as barriers to the integration of the app within physiotherapy sessions. Additionally, physiotherapists would have liked to see progress on the three modules displayed in the exact same way in the physiotherapist dashboard as in the app, so that when integrating the app within physiotherapy sessions, progress can be viewed in the patient’s app or in the physiotherapist dashboard.

*You have to dive into such a program, while the patient is sitting next to you. You don’t have a simple overview, a kind of cockpit, in which you can see where the patients stands. That would be very useful. [Physiotherapist 21]*

*Design quality of the patient app.* Patients found it pleasant that exercise instruction videos and information were all together in a single app. Content of the app was considered to be complete, but navigating through the app was a challenge for some patients. Additionally, patients were not always able to find their progress in the app. Therefore, patients suggested the creation of a table of contents or instruction video about the content and structure of the app.

*Even if it only contains a table of content, so to speak. Like ‘here you can find the exercises, here you can find information’ and besides that briefly explained what you can use it all for. … The moment that people read like ‘what’s in it for me’, then they will continue. [Patient 7]*

#### Paper-based workbook

*Added value.* Physiotherapists said they were interested in the best, fastest and easiest mode of treatment delivery. Physiotherapists agreed that the most added value was in the app. They also agreed that if the app is not suitable for a patient, the paper-based workbook would be a good alternative. Yet, none of the physiotherapists used the paper-based workbook, as they assessed there were no patients that were unsuitable for e-Exercise. An added value of the paper-based workbook they described was that it was relatively easy to point out which information themes need to be read by the patient.

*For people who will not succeed in using the app, you can do this just fine. [physiotherapist 20]*

#### Information module in the patient app

*Added values.* Physiotherapists stated that the information themes could really complement information provision provided in physiotherapy sessions. Integrating the information module within treatment sessions, helped the patient to progress from specific advice to action. A patient explained that by reading the information texts they started investigating what information relates to themselves. Thereafter, the patient was able to explore this in-depth with the physiotherapist within treatment sessions. Additionally the patient’s answer to an assignment helped the physiotherapist to provide insight in to the extent to which the patient understood the information.

*Because you read about it, you start investigating what relates to yourself, and because of that, you come to other points with your physiotherapist. You will also start asking more critical questions. [Patient 7]*

*Design quality.* Patients experienced the app’s figures and images as relatively small on some smartphones. Therefore they suggested to consider to create a zoom function. Additionally, patients mentioned they missed a search or ‘Questions & Answers’-function in the app, as one patient explained:

*When you have a question or there is something you are curious about, that you can dive into that a little bit deeper. You want information in the app, but most of the information is not applicable to you. [Patient 9]*

*Complexity.* One patient reported they did not have strong reading skills, but found the information texts in the app easy to follow and understand. However, some patients found the information texts too long. They suggested to show one key phrase to read directly, so they can read the longer text at the end of the day.

#### Exercise module in the patient app

*Added values.* Physiotherapists and patients both experienced the exercise module as providing added value. Patients recognized that the exercise module and corresponding reminders supported them in doing their home exercises and motivated them to go to the gym. Some patients reported that they continued doing the exercises after conclusion of their treatment, or showed the exercise videos to their fitness instructor, to add to the patient’s general fitness regime.

*It also motivates to start doing something like fitness. [Patient 10]*

*Design quality.* Out of the three modules, physiotherapists reported that the exercise module was the hardest to master. Physiotherapists explained that this was due to the separate system to set up exercises aside from the physiotherapist dashboard. A suggested solution was to practice this more intensively during the physiotherapists’ training.

*Adherence*. Patients studied the exercise instruction videos for the first few times whilst doing their exercises, to watch the exercise performance instructions. At some point, patients knew how to perform the exercises and explained they did not need the app anymore. Some patients then stopped with the exercise evaluations. However, some patients kept on using the app, because they knew that the physiotherapist wanted to see the evaluation. If patients received a message that new exercises were available, they all started using the app again.

*I always did the exercises with the app, because I had to indicate how well it went and whether I had done them all, and she could check that of course. So I did use the app. Yes. [Patient 4]*

#### Physical activity module in the patient app

*Added value.* Physiotherapists reported that the physical activity module was not applicable to every patient. They explained that most patients with neck and/or shoulder complaints are already physically active enough. In these cases, physical activity was not the main focus of treatment. Physiotherapists suggested to make the physical activity module an optional module. However, both physiotherapists and patients did not mind that the physical activity module was part of the app, since the decision to use it was optional.

*Design quality of the physiotherapist dashboard.* Some physiotherapists suggested to consider that the physical activity goal for patients is not chosen from a fixed list of activities. Instead, they preferred an open field so patients can choose a specific daily- or physical activity.

*These activities. I believe it would be clever to make that an open field. Because rowing, I do not know a lot of people who do that three times a week. For walking or cycling I do. But there are many more activities besides these options. [Physiotherapist 21]*

*Design quality of the patient app.* Patients mentioned that the stopwatch function of the physical activity module was a little outdated and they preferred an automatic physical activity tracker function. Additionally, when a patient accidently forgot the stopwatch or stopped it early, they experienced that the app provided incorrect feedback. Patients would have liked to add comments to explain why they were more or less physically active than their goal. Also, patients experienced that when they performed their physical activity on another day than planned, the app generated a message to discuss the reasons with a physiotherapist. One patient experienced that comment as demotivating.

*Actually, the app should be able to register whether you are walking or cycling. Now I can just sit at home and turn the stopwatch on. [Patient 1]*

## Discussion

In the first phase of this study, matched treatment options were developed for six subgroups as part of the Stratified Blended Physiotherapy approach for patients with neck and/or shoulder complaints. Recommendations about the content and intensity of physiotherapy treatment were matched to the patient’s risk of persistent disabling pain (using the Keele STarT MSK Tool; low, medium, or high risk), and mode of physiotherapy delivery was intended to be matched to the patient’s suitability for blended care (using the Dutch Blended Physiotherapy Checklist (i.e. yes or no). A paper-based workbook and the e-Exercise app modules for patients with neck and/or shoulder complaints were developed as practical tools for the mode of delivery matched treatment options. In the second phase of this study, feasibility of the developed Stratified Blended Physiotherapy approach for patients with neck and/or shoulder complaints was investigated for both patients and physiotherapists. Patients and physiotherapists were ‘a little’ satisfied with the new approach. Of the patients who were willing to participate, they considered the e-Exercise app as ‘best imaginable’ usability. Usability of the physiotherapist dashboard to set up the e-Exercise app was considered ‘OK’ by physiotherapists. This can be largely explained by the lack of a cockpit functionality with an overview of active patients and their progress and a lack of a logical workflow. The paper-based workbook was not used, so there is no data of patients that were unsuitable for blended care. Results of the second phase can be used to develop, improve or implement e-Exercise or other blended interventions.

This is the first study to develop and test feasibility of integrated risk stratification and blended care in patients with neck and/or shoulder complaints. Also, no stratification tools to identify patient subgroups and subsequently match them to a treatment are recommended by Dutch physiotherapy guidelines for neck/shoulder pain [32, 34, 35]. Our findings suggest that the Stratified Blended Physiotherapy approach might support physiotherapists with concrete stratification tools to provide patient-centered care. However, none of the physiotherapists in our study used the paper-based workbook as an alternative to the blended mode of treatment delivery e-Exercise. Therefore, there are no findings on the feasibility of the matched treatments that include the paper-based workbook. It is not known whether this can be explained by selection bias of patients that were suitable for e-Exercise by physiotherapists, whether the sample of physiotherapists was biased and were all eager to use e-Exercise, or whether there were no suitable patients. In the subsequent cluster randomized trial, a usual care arm will be the comparison and suitability for blended care will be monitored in both trial arms [20]. The other stratification tool was the Keele STarT MSK Tool. Our results show that the stratification tool was experienced as practically applicable. As for the recommended matched treatments, recently, recommended matched treatment options were developed for use in primary care treatment of MSK pain, including neck pain and shoulder pain [13]. These findings are consistent with the focus of our physiotherapy-specific matched treatment options for the Dutch neck and/or shoulder patient population.

In this study, we identified some improvements needed to be made to the Stratified Blended Physiotherapy approach before initiating a cluster randomized trial to determine the clinical effectiveness of the new approach compared to usual physiotherapy. First, patients needed an overview of the steps and possibilities of the e-Exercise app module. Therefore, an instruction sheet called ‘working with the e-Exercise program through MijnZorgApp’ was developed for patients. Second, physiotherapists needed a cockpit functionality in the physiotherapist dashboard of the app module with an overview of active patients and their progress. Therefore, a cockpit functionality was developed for physiotherapists to see which action is needed for which active patient, with the aim to provide a more logical workflow. Third, improvements need to be made to the physical activity module of the app. It is suggested to integrate an activity tracker or personal health environment with the app. The integration of an activity tracking device with a display was also suggested in a previously executed feasibility study on e-Exercise [23]. This cannot be accomplished before the execution of a cluster randomized trial, due to technical, budget and planning restrictions, but will be done thereafter.

A strength of this study was the use of knowledge from a previously developed and evaluated stratified blended intervention for patients with other MSK complaints, namely non-specific low back pain (e-Exercise LBP), in which patients and physiotherapists were involved in the participative development, and was conducted by the same research group [16, 23]. Also, the involvement of various stakeholders was valuable in the participative intervention development process. Participative development is recommended in the development of complex (eHealth) interventions and a model such as the CeHRes Roadmap can be used to guide the process [36, 37]. An advantage of the intervention was that the e-Exercise app module is integrated within one of the largest EHR systems of the Netherlands. However, physiotherapists with other EHR systems used a stand-alone version of e-Exercise and therefore had to use two independent systems. This might have impacted feasibility outcomes, but is also the reality within the Dutch Physiotherapy setting.

Despite the careful execution of this study, there are some methodological considerations. The digital questionnaire was sent three months after the first physiotherapy session. This is a relatively long time gap, especially for patients at low risk, which might have led to recall bias. However, we chose follow-up at 3 months to ensure that all participants would have been able to receive their matched intervention, and to ensure the follow-up time-point was the same for all subgroups. Also, because of the small number of included patients, physiotherapists did not gain the experience to fully familiarize the Stratified Blended Physiotherapy approach. Therefore, there were no physiotherapists which made the Stratified Blended Physiotherapy approach part of their daily work routine, which might have influenced feasibility outcomes. Experiences might have been different in that theoretical group, because behavior change of the physiotherapist will need time and experience. Additionally, no a priori sample size calculations were done. The small sample of physiotherapists and patients, and the fact that only one patient at high risk was included in the feasibility analyses might have led to limited generalizability. Therefore, in the subsequent cluster randomized trial, continuous actions will be taken to maximize inclusion rates.

## Conclusion

In conclusion, this study described the participative intervention development and feasibility testing of physiotherapy delivered matched treatment options as part of the Stratified Blended Physiotherapy approach for patients with neck and/or shoulder complaints. To serve a diverse group of patients, both app-based as well as paper-based materials were developed. However, the paper-based workbook was not used in the feasibility study. The results have informed amendments to the Stratified Blended Physiotherapy approach for patients with neck and/or shoulder complaints ready to use within a future cluster randomized trial.

## Electronic supplementary material

Below is the link to the electronic supplementary material.

Supplementary Material 1

## Data Availability

The datasets used and analyzed during the current study are available from the corresponding author on reasonable request.

## List of abbreviations

MSK: Musculoskeletal
STarT: Subgroup targeted treatment
UK: United Kingdom
GPE-DV: Global Perceived Effect – Dutch Version
NPS: Net Promoter Score
SUS: System Usability Scale
EHR: Electronic health record
LBP: Low back pain
