# Supplementary material for: Development and feasibility of stratified primary care physiotherapy integrated with eHealth in patients with neck and/or shoulder complaints: results of a mixed methods study
Source: BMC Musculoskelet Disord. 2023 Mar 9;24:176. doi: 10.1186/s12891-023-06272-6 (PMC9996840; doi:10.1186/s12891-023-06272-6)

**Appendix 1. Topic lists interviews phase 2**

| **Main theme** | **Sub theme** |
| --- | --- |
| *Patient topic list* |  |
| Characteristics of the patient | (1) Motivation for participation  (2) eHealth experience  (3) Content of treatment  (4) Number of treatment sessions  (5) Patient satisfaction  (6) Personal benefits  (7) Personal drawbacks  (8) Professional obligation  (9) Knowledge  (10) Awareness of content of e-Exercise |
| Characteristics of the intervention | (1) General impression e-Exercise  (2) Degree of app usage  (3) Relevance for patient  (4) Time investment  (5) Procedural clarity of app installation  (6) Procedural clarity of logging in  (7) Procedural clarity of the information module  (8) Procedural clarity of the exercise module  (9) Procedural clarity of the physical activity module  (10) Feedback given by the app  (11) Added value of e-Exercise  (12) Observability  (13) Completeness  (14) Complexity  (15) Language usage |
| Characteristics of the organization | (1) Integration of the web-based application within treatment sessions  (2) Quality and used interventions within treatment sessions |
| *Physiotherapist topic list* |  |
| Characteristics of the physiotherapist | (1) Motivation for participation  (2) eHealth experience  (3) Experience with prognostic risk stratification  (4) Patient recruitment  (5) Physiotherapist satisfaction  (6) Patient satisfaction  (7) Personal benefits  (8) Personal drawbacks  (9) Professional obligation  (10) Knowledge  (11) Awareness of content of e-Exercise |
| Characteristics of the intervention | (1) Physiotherapist training in e-Exercise  (2) Procedural clarity of the Stratified approach  (3) Confidence in the Stratified approach  (4) Keele STarT MSK Tool and matched treatments  (5) Dutch Blended Physiotherapy checklist and matched treatments  (6) General impression of e-Exercise and paper-based workbook  (7) Relevance of app and paper-based workbook for patient and physiotherapist  (8) Completeness of the app and paper-based workbook  (9) Degree of usage of the physiotherapist dashboard to set up the app and the paper-based workbook  (10) Integration of the app or paper-based workbook within treatment sessions  (11) Procedural clarity of e-Exercise  (12) Complexity of the app and paper-based workbook  (13) Compatibility of e-Exercise and the paper-based workbook |
| Characteristics of the organization | (1) Support by colleagues  (2) Time |

**Appendix 2. Photo of a person using one of the e-Exercise app modules**


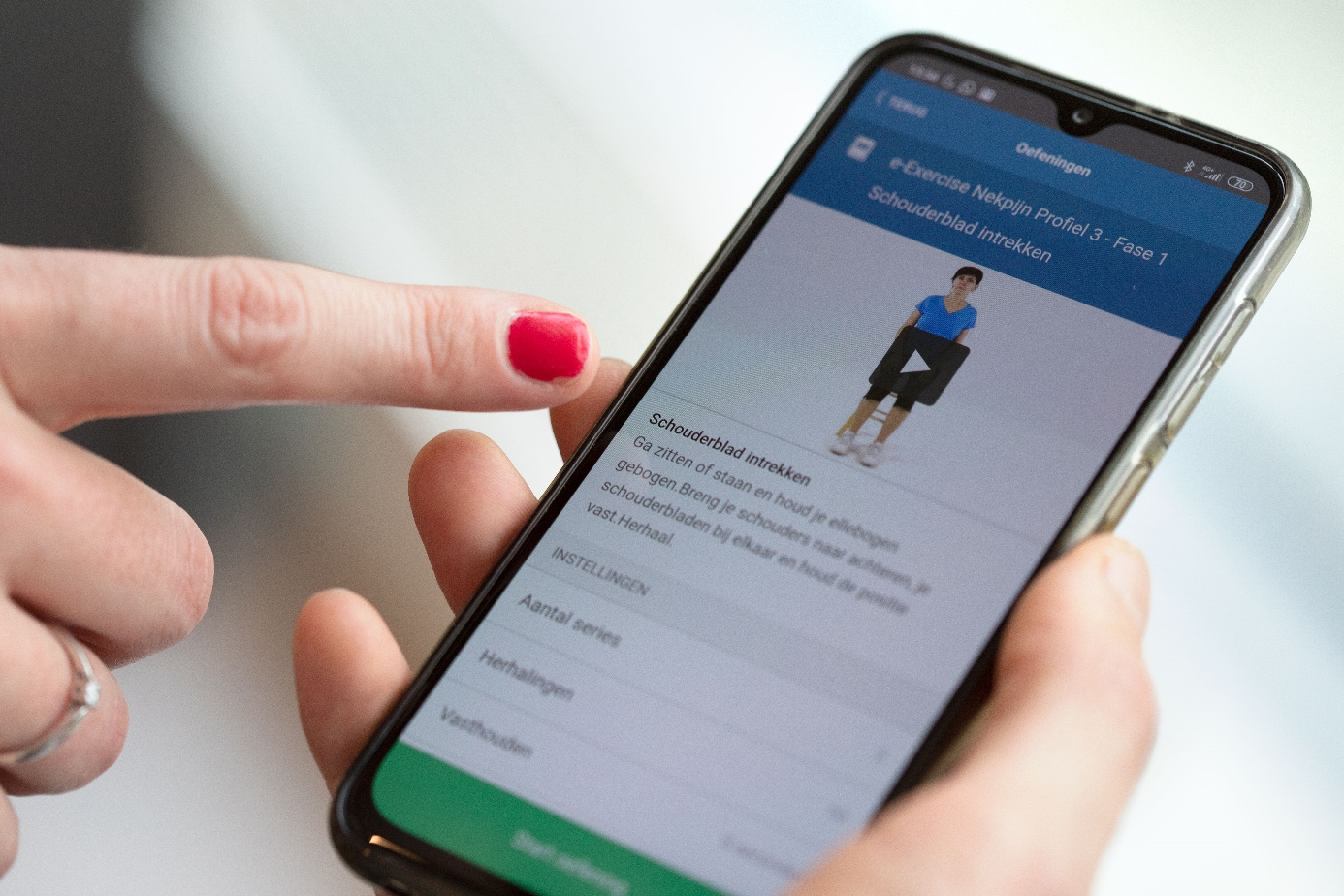

Supplement: Supplementary file 1 — Supplementary Material 1 [file 12891_2023_6272_MOESM1_ESM.docx]
